# Removal Capacities of Polycyclic Aromatic Hydrocarbons (PAHs) by a Newly Isolated Strain from Oilfield Produced Water

**DOI:** 10.3390/ijerph14020215

**Published:** 2017-02-22

**Authors:** Yi-Bin Qi, Chen-Yu Wang, Cheng-Yuan Lv, Zeng-Min Lun, Cheng-Gang Zheng

**Affiliations:** 1 Petroleum Exploration & Production Research Institute, SINOPEC, No. 31, Xueyuan Road, Haidian District, Beijing 100083, China; lcy.syky@sinopec.com (C.-Y.L.); lunzm.syky@sinopec.com (Z.-M.L.); zhengcg.syky@sinopec.com (C.-G.Z.); 2 China University of Geosciences-Beijing, College of Energy, No. 29, Xueyuan Road, Haidian District, Beijing 100083, China; wangchenyu1996@163.com

**Keywords:** polycyclic aromatic hydrocarbons, crude oil, *Gordonia*, degradation, bioremediation

## Abstract

The polycyclic aromatic hydrocarbon (PAH)-degrading strain Q8 was isolated from oilfield produced water. According to the analysis of a biochemical test, 16S rRNA gene, house-keeping genes and DNA–DNA hybridization, strain Q8 was assigned to a novel species of the genus *Gordonia*. The strain could not only grow in mineral salt medium (MM) and utilize naphthalene and pyrene as its sole carbon source, but also degraded mixed naphthalene, phenanthrene, anthracene and pyrene. The degradation ratio of these four PAHs reached 100%, 95.4%, 73.8% and 53.4% respectively after being degraded by Q8 for seven days. A comparative experiment found that the PAHs degradation efficiency of Q8 is higher than that of *Gordonia alkaliphila* and *Gordonia paraffinivorans*, which have the capacities to remove PAHs. Fourier transform infrared spectra, saturate, aromatic, resin and asphaltene (SARA) and gas chromatography–mass spectrometry (GC–MS) analysis of crude oil degraded by Q8 were also studied. The results showed that Q8 could utilize *n*-alkanes and PAHs in crude oil. The relative proportions of the naphthalene series, phenanthrene series, thiophene series, fluorene series, chrysene series, C21-triaromatic steroid, pyrene, and benz(a)pyrene were reduced after being degraded by Q8. *Gordonia* sp. nov. Q8 had the capacity to remediate water and soil environments contaminated by PAHs or crude oil, and provided a feasible way for the bioremediation of PAHs and oil pollution.

## 1. Introduction

Polycyclic aromatic hydrocarbons (PAHs) constitute a large and diverse group of priority environmental pollutants and can be formed as products during incomplete combustion of organic matter [1]. PAHs are widespread environmental pollutants commonly found in soil, surface waters, and sediments and their fate in nature is of great environmental concern due to their potential hazards of toxicity, mutagenicity, and carcinogenicity [2,3]. Recent research has shown that chronic exposure to PAHs is associated with cancerous diseases in aquatic animals and enhanced mutagenicity of sediments. Due to their toxicity, carcinogenicity, and ubiquitous distribution, the US Environmental Protection Agency has listed 16 PAHs as priority pollutants [4].

PAHs are natural constituents of fossil fuels and present in relatively high concentrations in petroleum products, so the petroleum industry and transport process are the major pathways which could produce PAHs in the environment [5]. PAHs can enter the environment in many pathways, such as volatilization, photo-oxidation, chemical oxidation, bioaccumulation, adsorption in soil particles and so on [6]. However, research has shown that microbial degradation, with a range of advantages compared to more traditional methods, has been developed as an effective technology for PAH removal [7]. Microbial degradation is a method of bioremediation, which consists of seeding microorganisms in polluted environments to achieve the aim of bioremediation, and it is considered a valuable tool for increasing the rate and extent of biodegradation of pollutants [8]. Nowadays, in order to eliminate PAHs from the environment by bioremediation, many PAH-degradation microorganisms have been isolated. Several articles have focused on the isolation and characterization of strains with the ability to grow using PAHs as their sole carbon and energy source, such as naphthalene, phenanthrene, fluoranthene and pyrene, and various bacteria capable of degrading PAHs were discovered [9,10]. Most of these bacteria belong to the genera *Pseudomonas*, *Rhodococcus*, *Paenibacillus*, *Acinetobacter*, *Bacillus* and *Mycobacterium* [11,12,13,14,15,16]. Recently, different aspects such as PAH metabolism of bacteria, degradation mechanisms, degradation related enzymes of PAHs and related catabolic genes have been researched [17,18,19].

Petroleum components have traditionally been divided into four fractions: saturated hydrocarbons, aromatics, resins, and asphaltenes [20]. Normally, saturated hydrocarbons, such as short-chain saturated hydrocarbons and low-molecular-weight aromatics, are the most susceptible to biodegradation, whereas PAHs in aromatics and resins are less vulnerable to microbial attack [21]. Therefore, screening for bacteria that could degrade PAHs in petroleum contaminated areas is an important part of bioremediation research [22].

According to previous studies, the genus *Gordonia* has the ability to degrade naphthalene, phenanthrene, anthracene, pyrene and PAHs in crude oil. The aim of this work was to isolate and characterize a newly PAH-degrading bacterium, *Gordonia* sp. nov. strain Q8, and to evaluate its PAH-degradation potential.

## 2. Materials and Methods

### 2.1. Sample Sources and Culture Media

Isolated samples, including water and crude oil, were collected from the Jiangsu Wei5 oilfield located in East China (east longitude 119°37′ and north latitude 32°58′). The injection water was recycled after oil–water separation of the production water from the oil wells. The depth of the petroleum reservoir is 1046–1138 m, with a temperature of 40 °C. The viscosity of the crude oil in the reservoir is 528 mPa·s. The air permeability of the reservoir is 0.330 μm^2^.

Growth of selected strains on PAHs was determined in mineral salt medium (MM) with the following composition (per liter): 1.0 g K_2_HPO_4_, 1.0 g KH_2_PO_4_, 2.0 g NaNO_3_, 0.5 g MgSO_4_·7H_2_O, 0.5 g (NH_4_)_2_SO_4_, 0.2 mg Na_2_MoO_4_ and the solution pH was adjusted to 7.0. Solid MM plate was prepared by adding 20.0 g agar into 1000 mL MM.

### 2.2. Microorganisms

Type strains: *Gordonia alkaliphila* JCM 18077T and *Gordonia paraffinivorans* DSM 44604T. The two strains are *Gordonia* type strains and provided by the German ZALF institute (Müncheberg, Germany).

### 2.3. Enrichment and Selection of PAH-Degrading Bacteria

The crude oil was added to the MM (2 g/L). The mixture was cultured in a shaking incubator at 40 °C until the oil was emulsified. Five percent of the enrichment liquid was then collected and inoculated with fresh MM, and cultured under the same conditions; this process was repeated at least three times before the bacterial strains were isolated. Isolation and purification procedures were carried out on MM agar plates by conventional spread plate techniques. 0.2% (*w*/*v*) resin was dissolved in hexane and sprayed on the surface of the pure culture as the sole carbon source. Each potentially different colony was selected from the MM agar plates. 18 strains, named Q1–Q18, were isolated from oilfield produced water. The strain Q8 was selected for further studies due to its ability to grow on solid media supplemented with naphthalene and pyrene.

### 2.4. Analysis of 16S rDNA Sequence

16S rDNA of strains Q8 were amplified by using universal eubacterial primers 8f (5′-AGAGTTTGATCCATGGCTCAG-3′) and 1541r (5′-AAGGAGGTGATCCAGCCGCA-3′) for bacterial fragments (≈1500 bp) from the total DNA. Polymerase chain reaction (PCR) amplification was performed in a 25 μL mixture containing 2.5 μL 10× Buffer, 2 μL dNTP mixture (0.2 mmol/L), 0.2 μL upstream primer (50 pmol/L), 0.2 μL downstream primer (50 pmol/L), 50 ng of purified DNA extract and 1 U Taq DNA polymerase. PCR cycle conditions were as follows: 5 min of initial denaturation at 94 °C, 30 cycles of 90 s denaturation at 94 °C, 2 min of annealing at 55 °C, followed by 2 min of extension at 72 °C and finally 10 min of extension at 72 °C before cooling to 4 °C [23].

### 2.5. Determination of GC Content and DNA–DNA Hybridization

GC content was determined by high-performance liquid chromatography [24], and calculated from the ratios of deoxyguanosine and thymidine. DNA–DNA hybridization to determine genomic relatedness was also performed [25]. Hybridization was performed with five replications for each sample. DNA–DNA relatedness values are the average of the five values.

### 2.6. Amplification and Sequencing of Housekeeping Genes alkB, catA, gyrB and secA1

Amplification and sequencing of the housekeeping genes *alkB*, *catA*, *gyrB* and *secA1* was performed by the method of PCR [26]. The genes were purified by gel electrophoresis. The purified genes were sent to Shanghai Meiji Biological Technology Company (Shanghai, China) and sequenced.

### 2.7. NCBI Accession Number

Partial 16S rRNA gene and housekeeping gene sequences for strain Q8 were deposited in GenBank databases under accession numbers 16S rDNA (KX539548), *alkB* (KX539549), *catA* (KX539550), *gyrB* (KX539551) and *secA1* (KX539552).

### 2.8. Biodegradation of PAHs by Strain

The ability of strains to degrade single PAHs and PAH compounds was determined in MM. Strains were cultured at 40 °C for 3 days under aerobic conditions (rotatory shaker, 150 rpm) with 0.1% *w*/*v* of sucrose. Overnight culture of each bacterial strain was harvested by centrifugation at 8000× *g* for 5 min and re-suspended in sterile phosphate buffer (150 nM, pH 7) to yield an optical density of 0.8 at 600 nm (2.4 × 10^8^ cfu/mL). Aliquots (2 mL) of the cell suspensions were transferred to 250 mL Erlenmeyer flasks containing 100 mL of MM amended with a single PAH, naphthalene or pyrene, at the concentration of 500 mg/L [27]. The cultures were shaken at 40 °C for 14 days. The PAH degradation efficiencies were detected by gas chromatography (Agilent 7810, Santa Clara, CA, USA). The concentration of strains was measured by spectrophotometer at 600 nm. The changes of the above data were measured at 0, 3, 7, 10 and 14 days [28]. Aliquots (2 mL) of the cell suspensions were transferred to 250 mL Erlenmeyer flasks containing 100 mL of MM amended with a mixture of four PAHs (naphthalene, anthracene, anthracene, and pyrene, each at a concentration of 500 mg/L). The cultures were shaken at 40 °C for 7 days. The PAH degradation efficiencies were detected at 3 and 7 days (Agilent 7810) [29].

### 2.9. Measurement of PAH Degradation in Petroleum

Aliquots (2 mL) of the cell suspensions were transferred to 250 mL Erlenmeyer flasks containing 100 mL of MM amended with 2 g of crude oil from the Wei5 oil well. The cultures were shaken at 40 °C for 14 days and then the petroleum was harvested by centrifugation at 10,000 rpm for 5 min. The petroleum was weighed after being dried and the degradation ratio was calculated [30].

The oil degraded by each strain was measured by Fourier transform infrared spectroscopy (FTIR) [3]. About 2 mg of the dried petroleum was carefully mixed with 300 mg of dry KBr and pressed into a self-supporting pellet. FTIR measurements were performed using a FTIR model FTS-40 (Bio-Rad, Hercules, CA, USA). For each sample, 32 spectrums were accumulated between 4000 cm^−1^ and 400 cm^−1^, and averaged.

Oil composition analysis of petroleum degraded by each strain was carried out [31]. After incubation, the culture was extracted sequentially with equal volumes of hexane, methylene chloride and chloroform. All extracts were pooled, dried at room temperature by evaporation in a safety cabinet and used as the residual crude oil. Then the residual crude oil was separated into alkane, aromatic hydrocarbon, resins and asphaltene fractions using a silica gel G (60–120 mesh) column (Sigma-Aldrich, Saint Louis, MO, USA) (2 cm × 30 cm). A series of PAHs in petroleum were analyzed according to the Chinese Standard SY/T 5779-2008 by gas chromatography–mass spectroscopy (GC–MS). The chromatographic column was a HP-5MS quartz capillary column (60 m × 0.25 mm × 0.25 μm) mounted in an Agilent 7890-5975c gas chromatography mass spectrometer. The GC oven was programmed at 50 °C for 1 min, from 50 to 120 °C at 15 °C/min, from 12 to 300 °C at 3 °C/min, and held at 300 °C for 25 min; helium with 1 mL/min velocity was used as the carrier gas. MS was conducted in full scan mode and the absolute voltage was 1047 V [32].

## 3. Results

### 3.1. Description and Identification of Strain Q8

Strain Q8, a bacterial isolate capable of degrading PAHs, was obtained from oilfield produced water. This strain formed smooth, wet, and convex colonies, which were slightly yellow and circular with a diameter of 0.5–1 mm within 2–3 days. The colony could be easily scraped off of nutrient agar plates incubated at 40 °C for 2–3 days. Strain Q8 is a gram-positive, rod-shaped bacterium. The size of the bacteria is 1.0–4.0 μm × 0.5–1.2 μm, with an optimum growth temperature at 40 °C and no growth at 10 or 60 °C.

**Figure 1:**
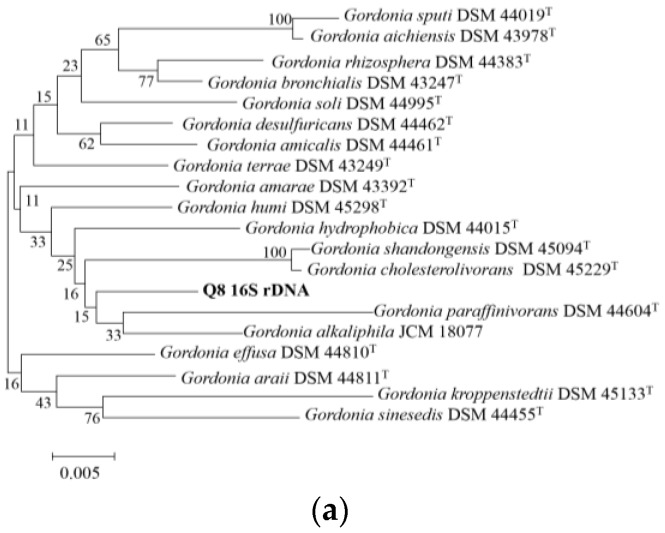

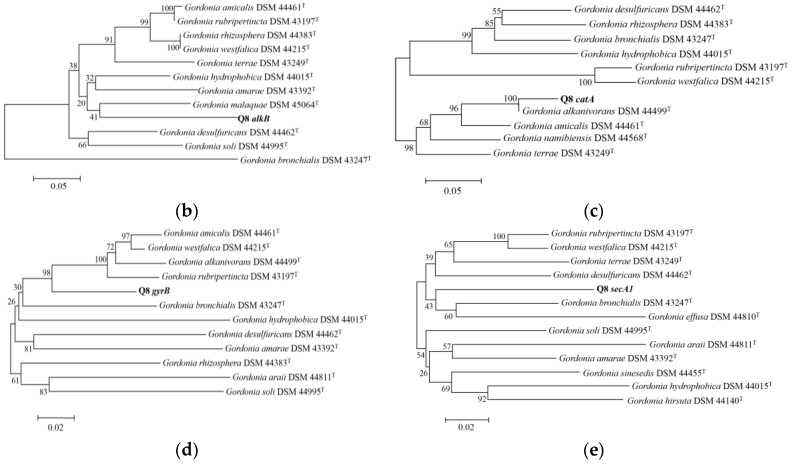
Phylogenetic trees of *Gordonia* sp. nov. Q8 with closely related sequences from the GenBank database based on 16S rRNA, *alkB*, *catA*, *gyrB* and *secA1* gene sequences. The scale bar represents inferred substitutions per nucleotide position. Values and nucleotide sequence accession numbers are also presented. (**a**) Phylogenetic tree of *Gordonia* sp. nov. Q8 based on 16S rRNA gene sequences; (**b**) Phylogenetic tree of *Gordonia* sp. nov. Q8 based on *alkB* gene sequences; (**c**) Phylogenetic tree of *Gordonia* sp. nov. Q8 based on *catA* gene sequences; (**d**) Phylogenetic tree of *Gordonia* sp. nov. Q8 based on *gyrB* gene sequences; (**e**) Phylogenetic tree of *Gordonia* sp. nov. Q8 based on *secA1* gene sequences.

The 16S rDNA and housekeeping gene sequencing were submitted to NCBI database for comparison, and the model strain with the highest homology sequence about the aligned sequences was selected. Based on the neighbor-joining method and Jukes & Cantor’s one parameter model, 16S rDNA and housekeeping gene phylogenetic trees of strain Q8 (Figure 1) were constructed [33,34].

Phylogenetic trees (Figure 1) showed that the similarity of the 16S rRNA gene sequence between strain Q8 and *Gordonia alkaliphila* JCM 18077T is 98.06%, and the similarity of the 16S rRNA gene sequence between strain Q8 and *Gordonia paraffinivorans* DSM 44604T is 97.43%. However, the colony and degradation ability of strain Q8 is different from other types of *Gordonia*, and the four housekeeping genes (*alkB*, *catA*, *gyrB* and *secA1*) of strain Q8 shared less than 89% similarity with other type strains of *Gordonia* (Figure 1). Further analysis indicated that the DNA base compositions of strain Q8, JCM 18077T and DSM 44604 were 66 mol %, 72 mol % and 61 mol % G + C respectively. The mean level of DNA–DNA relatedness between strains Q8 and JCM 18077T was 43.6%, and the mean level of DNA–DNA relatedness between strains Q8 and DSM 44604T was 47.6%. According to the recommendation of the International Committee on Systematic Bacteriology (ICSB), the lowest standard to determine a new species is the ratio of DNA homology ≤70%, thus strain Q8 was assigned to the genus *Gordonia* sp. nov. [35].

### 3.2. Growth with a Single PAH as the Sole Source of Carbon and Energy

**Figure 2:**
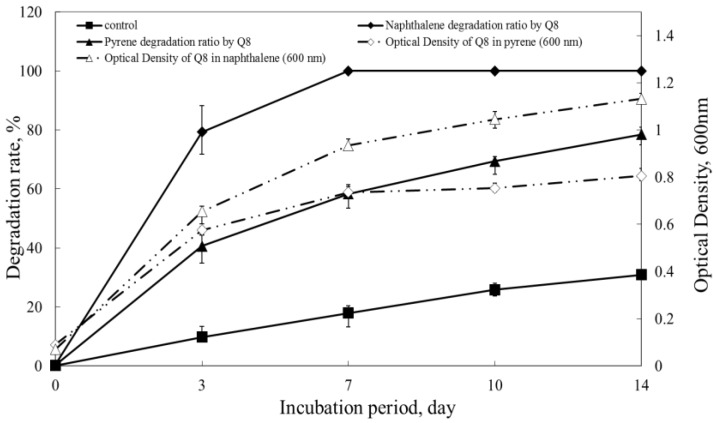
Growth of strain Q8 in mineral salt medium (MM) with naphthalene (△) and pyrene (◇) and percentage of naphthalene (◆) and pyrene (▲) degradation. Control was percentage of naphthalene evaporation (■) performed by inoculating with dead cells. Values are mean ± standard deviations of three replicates.

The results of GC analyses show that *Gordonia* sp. nov. Q8 could nearly degrade all naphthalene within 7 days, and degraded 78.5% pyrene within 14 days (Figure 2). Due to the volatilization of naphthalene, it has reduced gradually in control groups. However, the degradation ratios of Q8 were still higher than the control groups. The result indicated that *Gordonia* sp. nov. Q8 could grow with PAHs and degrade PAHs rapidly.

### 3.3. Biodegradation of PAHs

Many *Gordonia* strains capable of degrading PAHs have been isolated. Papers have reported that *Gordonia paraffinivorans* and *Gordonia alkanivorans* could degrade PAHs [36,37]. In this study, *Gordonia* sp. nov. Q8 could remove 100% of naphthalene in 7 days, whereas the degradation ratios of naphthalene by *Gordonia paraffinivorans* and *Gordonia alkanivorans* were less than 90% in 7 days. In the same conditions, Q8 could degrade phenanthrene, anthracene and pyrene faster than *Gordonia paraffinivorans* and *Gordonia alkanivorans* (Table 1). It is clear that strains that can degrade PAHs completely and rapidly will be more favored [38].

### 3.4. PAH Degradation in Crude Oil

The capacity of oil (collected from Jiangsu oilfield, with an original viscosity of 528 mPa·s) viscosity reduction and biodegradation by strains Q8 were also determined. The viscosity of petroleum decreased by 70.8% after being degraded by Q8 for 7 days, and the degradation rate reached 138 mg/day. The petroleum emulsified and dispersed in the biodegradation system indicated that crude oil could dissolve in water and was easy to utilize [38,39,40].

**Figure 3:**
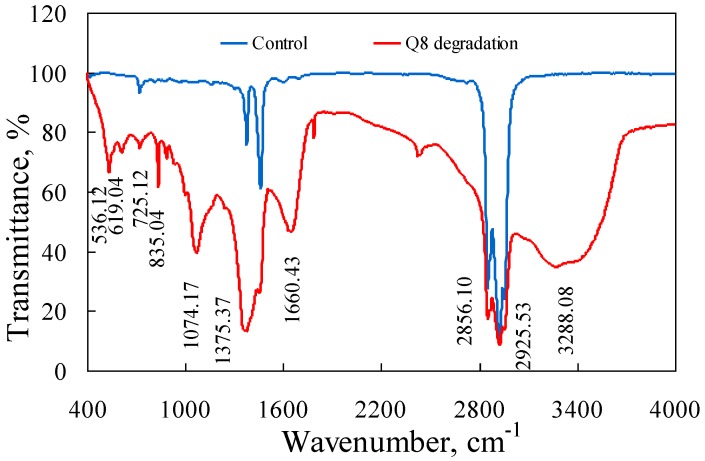
Fourier transform infrared spectra of Jiangsu Wei5 crude oil after degradation by Q8.

To investigate the oil degradation by strain Q8, several experiments were conducted to analyze the result of oil degradation. The FTIR spectra of the petroleum degraded by Q8 are shown in Figure 3. As absorption features from different components were superimposed in the fingerprint region, a detailed interpretation of the absorption bands was rendered difficult. After degradation, a large and wide absorption peak at 3405.38 cm^−1^, which originated from the stretching vibration of the hydrogen-bonded O–H, indicated the increase of oxidized oil. An absorption peak at 1660.43 cm^−1^ was significantly higher than the control, and this peak is the characteristic absorption peak of benzene. The change indicated that PAHs were degraded into benzene. In addition, a series of absorption peaks appear in the range of 700 to 1300 cm^−1^, indicating that many groups, such as hydroxyl and methoxyl, were produced during the process of oil degradation [41].

**Figure 4:**
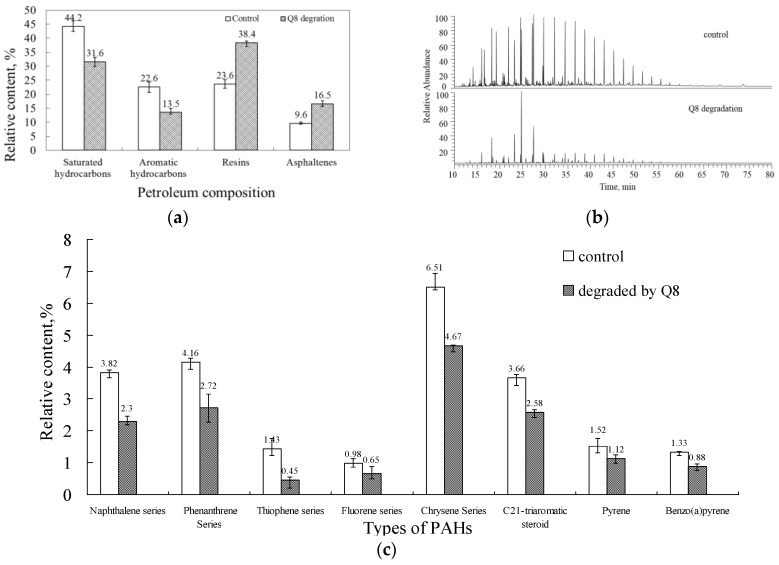
Differences analysis for items of oil degraded by Q8. The control represented the crude oil in the sterilized same condition. (**a**) Degradation of fractions of crude oil. Data are expressed as mean value and standard deviation of independent duplicates; (**b**) Gas chromatograms of total hydrocarbon fractions; (**c**) Biodegradation of the naphthalene, phenanthrene, thiophene, fluorene, and chrysene series, C21-triaromatic steroid, pyrene, and benzo(a)pyrene during biodegradation by Q8.

The results of the SARA (saturates, aromatics, resins, and asphaltenes) and GC–MS analysis of crude oil were shown in Figure 4 [42]. In order to ensure the reliability of the results, the total recoveries of the four components should be more than 90%. The results showed that the entire range of *n*-alkanes was degraded almost completely and the oil fractions varied greatly with saturated hydrocarbons decreased to 31.6% and aromatic hydrocarbons decreased to 13.5%, while the resins and asphaltene fractions increased by 14.8% and 6.9%, respectively.

**Figure 5:**
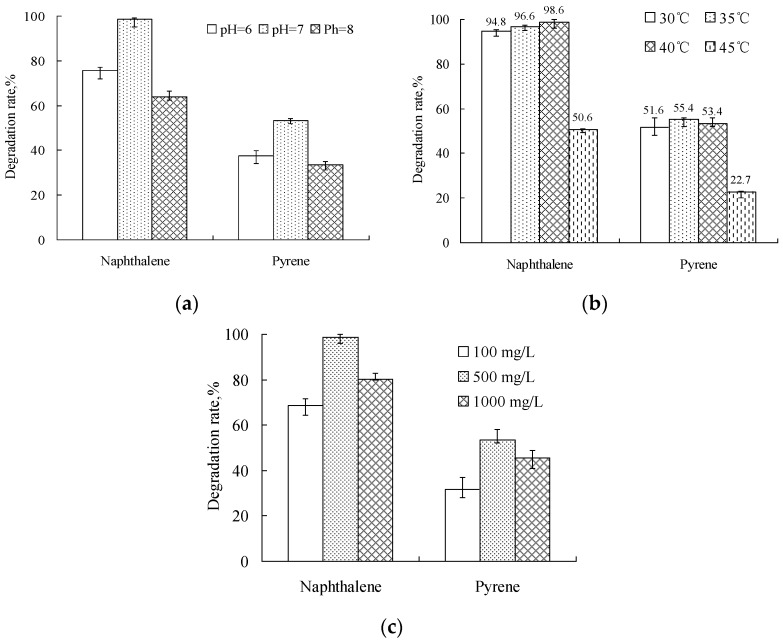
Effects of condition changes on biodegradation of polycyclic aromatic hydrocarbons by *Gordonia* sp. nov. Q8: (**a**) pH; (**b**) temperature; (**c**) initial concentration. Values are mean ± standard deviations of three replicates.

Microbial degradation of aromatic hydrocarbons was also investigated by GC–MS analysis (Figure 5). GC–MS analysis of the PAHs revealed that the relative contents of the naphthalene, phenanthrene, thiophene, fluorene, and chrysene series, C21-triaromatic steroid, pyrene, and benzo(a)pyrene decreased after degradation by Q8 [32].

## 4. Discussion

### 4.1. Advances in Bioremediation of PAHs

At this stage, there are several hot directions related to this article. Further studies may include screening more efficient and adaptable microorganisms with the capabilities to grow in extreme pH and in the presence of heavy metals and extreme environmental resistance to degrade PAHs [43]; researching factors including temperature, pH, oxygen, nutrients, excess substrates and end products that could affect the bioremediation of PAHs [13,44]; and researching the mechanism of co-metabolism and microbial consortia that could degrade PAHs completely. It has been observed that co-metabolism of one PAH could have a synergistic effect on the degradation of other PAHs, specifically for the degradation of high molecular weight PAHs [45,46]. Construct genetically engineered microorganisms: Using genetic engineering it is possible to enhance the activity or broad substrate specificity of certain enzymes associated with PAH-degrading pathways, which in turn will improve the mineralization of those pollutants in the environment [47].

### 4.2. Screening Efficient and Adaptable Bacteria with Capacities to Degrade PAHs

Recent studies have reported that chronic exposure to PAHs is associated with cancerous diseases and enhanced mutagenicity of sediments [48]. Due to their toxicity, carcinogenicity, and ubiquitous distribution, the removal of PAHs in the environment has received increasing attention from researchers [49]. Most species of microorganisms have the ability to degrade PAHs. The vast majority of organic matter in the environment can be degraded by microorganisms in nature. Studies have shown that more than 70 genera could degrade hydrocarbons [50]. Bioremediation of waste materials, which contain hydrocarbons and their derivatives, is based on the ability of microorganisms to increase their biomass growing on these substrates and degrading them to non-toxic products, such as H_2_O and CO_2_ [40]. So, the bioremediation of PAH-contaminated aquatic and soil environments has arisen as an effective technology, with a range of advantages compared with more traditional methods. Screening microorganisms from PAH-contaminated environments and then inoculating them into PAH-polluted environments is an important part of PAH bioremediation. Therefore, screening for efficient and diverse microorganisms is considered to be the foundation of PAH bioremediation [51,52].

Previous studies have reported that PAH-degrading strains mainly belong to the genera *Mycobacterium*, *Sphingomonas*, *Pseudomonas*, *Bacillus*, and *Cycloclasticus* [53,54]. In this study, strain Q8 was identified as a novel species of the genus *Gordonia* and the results showed that this strain is able to degrade PAHs faster than known *Gordonia* which have the capacity to degrade PAHs. Therefore, this study provides a new way for the bioremediation of PAHs.

### 4.3. Effect of Environmental Conditions on the Degradation of PAHs

Further degradation tests were carried out at various pH levels from 6.0 to 8.0, temperatures from 30 to 45 °C, and initial concentrations of each naphthalene and pyrene from 100 to 1000 mg/L. As shown in Figure 5a–c, the optimal conditions were determined to be at pH 7.0, 30~40 °C and 500 mg/L after 7 days incubation. The result also indicated that the degradation rate of strain Q8 decreased when the initial concentration of each kind of PAH is low (100 mg/L) or high (1000 mg/L). This is mainly because a lower PAH concentration is not enough for supporting the growth of strain Q8. On the other hand, a higher PAH concentration will lead to an increase of PAH metabolites’ toxicity. So, the PAH-degradation capabilities of strain Q8 can be exploited further for the development of effective bioremediation technology for environmental cleanup.

Bioremediation has an edge over other treatment methods because it can efficiently destroy the pollutant hydrocarbons present and does not allow the contaminant to accumulate [55]. The present studies showed that using the strain Q8, which can efficiently degrade crude oil components, maximum degradation was achieved at a temperature of 30~40 °C and pH of 7.0. Hence we suggest the use of the above optimized conditions and the strain for the bioremediation of crude oil-contaminated sites.

### 4.4. PAH Degradation in Oily Sludge and Sewage

In previous studies of PAH biodegradation, the tests concerning the biodegradation ratio of PAHs usually involved PAH-polluted soil. However, with the development and transportation of crude oil, soil and water may be contaminated by oil, and research has indicated that oily sludge and sewage have high concentrations of PAHs. So, degradation of PAHs in petroleum-contaminated sediment is an important part of reducing PAH pollution [38]. Therefore, it is necessary to characterize the performance of the strain in crude oil, and provide data for the bioremediation of oily sludge and sewage.

## 5. Conclusions

In the present study, strain Q8 (*Gordonia* sp.) was isolated from oilfield produced water. The degrading strain exhibited the following characteristics compared to former strains: (i) according to analysis of morphological observation, physiological and biochemical test, strain Q8 was assigned to a novel species of the genus *Gordonia*, and contrast experiments found that the PAH degradation efficiency of Q8 is higher than other types of *Gordonia*; (ii) Q8 could not only utilize naphthalene and pyrene as its sole carbon source, but also degraded mixed PAHs, and exhibited very high PAH degradation compared to known PAH-mineralizing bacteria reported so far; (iii) Q8 has a wide range of degradation performance, and is capable of PAH degradation in a wide range of temperatures; (iv) Q8 could utilize *n*-alkanes and a wide range of PAHs in crude oil. These results will provide an efficient method to circumvent the risk of PAH-contaminated sludge and sewage and may prove to be promising microorganisms for bioremediation to remove PAH-containing pollutants from contaminated sites.

## Figures and Tables

**Table 1:** Naphthalene, phenanthrene, anthracene, and pyrene removal (%) in mineral salt medium inoculated with *Gordonia* sp. nov. Q8, *Gordonia alkaliphila* JCM 18077T and *Gordonia paraffinivorans* DSM 44604T.

Strain	Naphthalene		Phenanthrene		Anthracene		Pyrene	
	72 h	168 h	72 h	168 h	72 h	168 h	72 h	168 h
Control	7.9 ± 0.2	18.6 ± 0.4	5.8 ± 0.2	6.6 ± 0.1	2.3 ± 0.1	3.9 ± 0.2	0.6 ± 0.3	0.8 ± 0.5
Q8 removal ratio (%)	75.6 ± 2.8	100 ± 0	45.9 ± 4.8	95.4 ± 4.6	40.4 ± 0.2	73.8 ± 2.5	37.7 ± 0.5	53.4 ± 0.1
18077T removal ratio (%)	38.5 ± 4.6	70.5 ± 2.1	31.5 ± 0.8	65.3 ± 2.2	28.6 ± 4.2	45.3 ± 1.1	18.6 ± 3.0	38.7 ± 0.6
44604T removal ratio (%)	48.5 ± 3.7	89.8 ± 0.6	42.7 ± 0.8	66.7 ± 1.7	33.6 ± 2.4	42.3 ± 0.2	28.6 ± 4.5	34.5 ± 2.8

Values are mean ± standard deviations of three replicates.
